# Mixed vaginal infection status in women infected with Trichomonas vaginalis: comparison of microscopy method and metagenomic sequencing analysis

**DOI:** 10.3389/fcimb.2025.1638464

**Published:** 2025-12-03

**Authors:** Li Jun, Xiulin Wan, Dai Zhang, Yan Zheng, Xi Chen, Lan Mi, Bingbing Xiao

**Affiliations:** 1 Ob&Gyn Department, Peking University First Hospital, Beijing, China; 2 Genome Analysis Laboratory of the Ministry of Agriculture and Rural Affairs, Agricultural Genomics Institute at Shenzhen, Chinese Academy of Agricultural Sciences, Shenzhen, Guangdong, China; 3 Department of Research and Development, Guangdong Research Institute of Genetic Diagnostic and Engineering Technologies for Thalassemia, Hybribio Limited, Guangzhou, China

**Keywords:** Vaginal trichomoniasis, mixed vaginitis, vaginal microbiome, community state type (CST) classification, metagenomic sequence

## Abstract

Trichomonas vaginalis (TV) infection is a common non-viral sexually transmitted infection, often combined with mixed vaginal infections. These mixed infections worsen inflammation, disrupt vaginal microbiota, and affect treatment. Currently, TV and its mixed infections are mainly diagnosed by wet mount microscopy, which has low sensitivity and cannot identify complex microbes well. This study compared microscopy with metagenomic sequencing to explore vaginal microbiota changes and improve diagnosis of TV-related mixed infections. We enrolled 30 participants: 20 TV-infected patients (diagnosed by wet mount microscopy) and 10 healthy controls (with Lactobacillus as dominant vaginal microbiota). Then tested by Gram staining, microscopy, and metagenomic sequencing. We analyzed microbial composition and identified different abundant taxa. We also measured clinical indices (Lactobacillus grade, vaginal pH, Nugent score for BV, Donders score for AV) to assess vaginal microecology. Among 20 TV patients, microscopy and clinical criteria found a 65% mixed infection rate (13/20), including TV+AV (5 cases), TV+BV+AV (7 cases), and TV+VVC (1 case). Metagenomic sequencing showed TV patients had higher alpha diversity (Shannon index: p=0.0276) and different beta diversity (ANOSIM, r=0.21, p=0.000167) than controls. At the genus level, TV patients had more anaerobic taxa (Fannyhessea, Atopobium, Peptostreptococcus, FDR<0.05) and less Lactobacillus (FDR<0.05) than controls. All TV patients were CST IV (low Lactobacillus, high mixed bacteria), including 12 cases of CST IV-C and 7 cases of CST IV-B. Microscopy and sequencing had low diagnostic consistency in diagnosing mixed infections, especially for mixed vaginitis. TV infection causes significant vaginal microecological imbalance (less Lactobacillus, more anaerobes, high mixed infection rate). Metagenomic sequencing is better than microscopy at identifying complex microbes and low-abundance pathogens, making it more accurate for diagnosing TV-related mixed infections. These results suggest molecular diagnostic methods should be used as complementary tools for precise analysis improve TV and its mixed infection diagnosis and treatment.

## Introduction

Vaginal trichomoniasis, a sexually transmitted disease (STD) caused by Trichomonas vaginalis (TV), is mainly transmitted through sexual contact but can also be acquired via indirect exposure. Trichomonas obtains nutrients by adhering to vaginal mucosal cells and releases proteolytic enzymes that damage local tissues, triggering inflammation. TV infection is associated with various adverse reproductive health outcomes, including increased risks of adverse birth outcomes (low birth weight, preterm labor, premature rupture of membranes), HIV and other sexually transmitted infections (STIs), pelvic inflammatory disease, infertility, and cervical cancer (Van Gerwen et al., 2021). Trichomoniasis is typically diagnosed via wet film microscopy, where motile trichomonads are observed.

Mixed vaginitis refers to simultaneous infection by two or more pathogens that cause corresponding clinical symptoms and signs. It is characterized by atypical symptoms, prolonged duration, and high recurrence rates, with a lack of precise diagnostic criteria—some vaginal inflammations cannot be categorized due to failure to meet existing criteria. Mixed infection is defined as concurrent pathogenic processes mediated by at least two vaginal pathogens, leading to vaginal inflammation with clinical manifestations. This differs from vaginal coinfections, where one pathogen causes symptoms while another is asymptomatically present, requiring treatment only for the symptomatic pathogen. Existing clinical criteria consider mixed vaginitis diagnosed when a patient meets the diagnostic criteria for two or more types of vaginitis with concurrent symptoms and signs of each. It’s common infection types include: the mixed infection of bacterial vaginosis (BV) and vulvovaginal candidiasis (VVC) (presenting symptoms of both conditions, such as vaginal discharge that is both homogeneous and thin as well as curd-like or tofu-like); aerobic vaginitis (AV)-related mixed infections (e.g., AV+BV, AV+VVC, and AV+TV, mostly accompanied by increased vaginal pH, mucosal inflammation, and yellow discharge); mixed infections of trichomonas vaginalis (TV) with other vaginitis (e.g., with symptoms including yellow-green discharge of TV and positive amine test of BV); and the relatively rare mixed infection of multiple pathogens (e.g., BV+VVC+TV, which is characterized by complex conditions and high treatment difficulty).

The global incidence of TV is approximately 156 million cases annually, with a prevalence of about 5.3% among women aged 15–49 years (Patel et al., 2018). Studies have shown that TV infection is associated with age, race, education, economic status, and number of sexual partners (Zheng et al., 2022). Zhang et al. reported a 2.83% prevalence of TV among rural reproductive-aged women in Beijing, China (Zhang et al., 2007), while Chen et al. found a 1.20% prevalence among women in Shantou, China (Chen et al., 2019). A global study on (female sex workers showed a 16% TV prevalence (Mirzadeh et al., 2021). Low-income countries have relatively high prevalence rates: recent data indicate 2.8–3.1% among women in North America (Cenkowski et al., 2022), higher rates in Black populations compared to other ethnic groups (Patel et al., 2018), and 9.2% among pregnant women in Africa (Gerson et al., 2020). The most common mixed vaginitis is bacterial vaginosis (BV) +vulvovaginal candidiasis (VVC) (20.95%-74.89%), followed by TV+BV (37.8%) and TV+BV+VVC (4.1%) (Qi et al., 2021).

Currently, the specific community composition, functional properties, and vaginal microbiota characteristics of TV-related mixed vaginitis remain unclear. In this study, we recruited patients infected with TV (both single and mixed infections) as well as patients without vaginitis to analyze vaginal microbiome characteristics in TV-infected women. The findings will enhance understanding of vaginal microbiome changes in TV-infected patients, assess the likelihood of mixed vaginitis in TV cases, and assist in treating TV infections and improving prognosis.

## Materials and methods

### Study design and data collection

The study protocol was approved by the Ethics Committee of the First Hospital of Peking University (Sample Bank Ethics Approval No. 2015–886 and Research Ethics Approval No. 2024 Research 372-002) and conducted in accordance with the Declaration of Helsinki. Twenty women diagnosed with TV infection by wet-film microscopy, and ten women without vaginitis (with Gram-positive large bacilli as the dominant vaginal microbiota) were selected from the reproductive tract infection sample bank. Participants were recruited from the gynecology outpatient clinic of the First Hospital of Peking University between January 2017 and November 2023. Vaginal microbiota were analyzed using metagenomic sequencing, and differential microbiota were identified via LEfSe analysis. Results were integrated with clinical diagnoses and symptoms, and microscopy findings were compared with metagenomic sequencing results to draw conclusions.

Sample bank specimen retention: Three sterilized swabs were rotated in the upper one-third of the posterior vaginal vault for sampling. One swab was used for wet slide microscopy. The second swab was used for Gram staining of vaginal secretions. The third swab was placed into a sterile labeled 2 ml Eppendorf tube and immediately frozen at -80 °C for vaginal microbiome analysis.

Inclusion criteria for sample bank: 1) Non-pregnant reproductive-aged women; 2) No vaginal bleeding; 3) No sexual intercourse, vaginal medication, or vaginal manipulation within 3 days; 4) No antibiotics within 1 month; 5) No oral contraceptives or other sex hormones within 3 months.

Due to limited research, there are no internationally standardized diagnostic or treatment criteria for mixed vaginitis. Chinese Clinical Diagnostic Criteria for vaginitis (Cooperative Group of Infectious Disease, 2021a):

1. TV: Detection of motile trichomonads in vaginal secretion suspensions via microscopic examination.
2. VVC: Detection of pseudohyphae or budding spores in Gram-stained secretion smears.
3. BV: Nugent score ≥7 in Gram-stained vaginal secretion smears.
4. AV: Donders score ≥3 with at least 1 clinical feature (e.g., vaginal mucosal congestion, yellow discharge) (Dong et al., 2021).

Chinese diagnostic criteria require mixed vaginitis to meet the diagnostic criteria for two or more types of vaginitis with concurrent symptoms and signs of each.

Vaginal microecological diagnostic criteria: The most abundant bacteria observed microscopically are defined as dominant microbiota. Dominant Gram-positive large bacilli (*Lactobacillus*-like) indicate normal microbiota; dominant Gram-negative small bacilli (*Gardnerella Vaginalis*-like) indicate abnormal BV-type microbiota; absence of dominant bacteria indicates microbiota suppression; other dominant bacteria indicate non-BV-type microbiota abnormalities.

TV-infected patients were subgrouped by co-infection type: TV+VVC, TV+BV, and TV+AV.

### Metagenomic sequencing and bioinformatics processing

Genomic DNA was extracted from vaginal secretion samples using the GensKey DNA Kit (GensKey, 2005-01) following the manufacturer’s instructions. DNA degradation and contamination were assessed via 1% agarose gel electrophoresis. DNA concentration was quantified using Qubit 3.0 and Nanodrop One instruments (Thermo Fisher Scientific, Waltham, USA). Metagenomic sequencing was performed by Guangzhou Hybribio Diagnostics Co., Ltd. (Guangzhou, China) on the BGISEQ platform, generating 50 bp single-end reads. Initial data quality inspection was performed with FastQC. Using DNBSEQ™ core technology, DNA nanoballs (DNBs) were loaded and immobilized on a regular array carrier via the instrument enterprise system, and then the sequencing template and sequencing reagents are pumped in. The pumped sequencing template hybridizes with the complementary junctions of the DNBs on the carrier sheet, and the sequencing template binds to the fluorescently labeled probes in the sequencing reagents under the catalysis of DNA polymerase. Gene libraries were sequenced single-end using the MGISEQ-200RS Metagenomic Sequencing Reagent Kit (FCL SE50), with fragments to be tested of approximately 300–350 bp, and a total sequencing read length of 71 bp, including 50 bp of the fragment to be tested and 10 bp of each of the double junctions.

QIIME (version 1.9.1, http://qiime.org/) was used to define Operational Taxonomic Units (OTUs) with >97% sequence homology. The OTU table of raw counts was normalized to relative abundance. Taxa were classified at phylum, class, family, and genus levels using databases including NCBI RefSeq, NCBI GenBank, FDA-ARGOS, and Genome Taxonomy Database. LEfSe analysis was performed to identify differentially abundant taxa between groups, with a threshold of LDA score > 3.0. This research aims to screen for highly associated biomarkers, the threshold was selected based on biological relevance (prioritizing taxa with substantial abundance differences (Nicola et al., 2011)and consistency with established practices in microbial community studies to avoid overinterpreting trivial variations. Beta diversity (between-sample differences) was evaluated using principal component analysis (PCA), principal coordinate analysis (PCoA), and non-metric multidimensional scaling (NMDS). Alpha diversity was assessed using the Shannon index at the OTU level, with differences between groups compared using the Wilcoxon rank-sum test. Shotgun metagenomic sequencing (untargeted sequencing of all microbial genomes in a sample) was used to analyze taxonomic composition, functional potential, and recover whole-genome sequences. Compared to 16S rRNA sequencing, metagenomic sequencing covers a broader range of genomic sequences with greater depth, identifies more bacterial species, and detects organisms from other kingdoms (viruses, fungi, protozoa), enabling enhanced diversity detection, gene prediction, and higher species identification accuracy. , 

### Statistical analysis

Data were analyzed using SPSS 27.0 statistical software and R package (v4.2.1). The main method of statistical analysis was descriptive analysis, and the mean ± standard deviation (x ± s) was used to indicate the age of the personnel and other indicators, and the count data were expressed as cases (%). Trends were analyzed using the χ^2^ test for trends. For multiple comparisons involving flora abundance at genus and species levels, the Benjamini-Hochberg method was applied to calculate the false discovery rate (FDR), and a corrected P value (FDR) < 0.05 was considered statistically significant. Changes in vaginal pH and Nugent scores were analyzed using the t-test (both two-sided tests).One-way ANOVA analysis with Tukey’s HSD *post-hoc* test was used to analyze the abundance of flora with different degrees of oxygen consumption and the abundance of flora with different morphologies.

## Results

### Grouping information, clinical characteristics and microscopic findings of the study population

Among 20 TV-infected patients, microscopic examination and clinical diagnostic criteria identified 7 cases of single TV infection, 1 case of TV+VVC, 5 cases of TV+AV, and 7 cases of TV+BV+AV. The mixed infection rate was 65% (13/20). Analysis of Gram-stained vaginal secretion smears showed a high mixed infection rate in TV-infected patients, accompanied by increased vaginal microbiota diversity, microbiota abnormalities, altered Lactobacillus grades, pH, Nugent score, and AV scores—with statistically significant differences between the infected and control groups (**Table 1**). The main clinical manifestations of TV-infected patients included pruritus, vaginal wall congestion, increased discharge, and foamy leukorrhea. Microscopic examination revealed a significant reduction in Lactobacillus, diverse dominant bacteria, and a high proportion of Gram-negative bacilli in the vaginal microbiota of TV-infected women.

**Figure 1:**
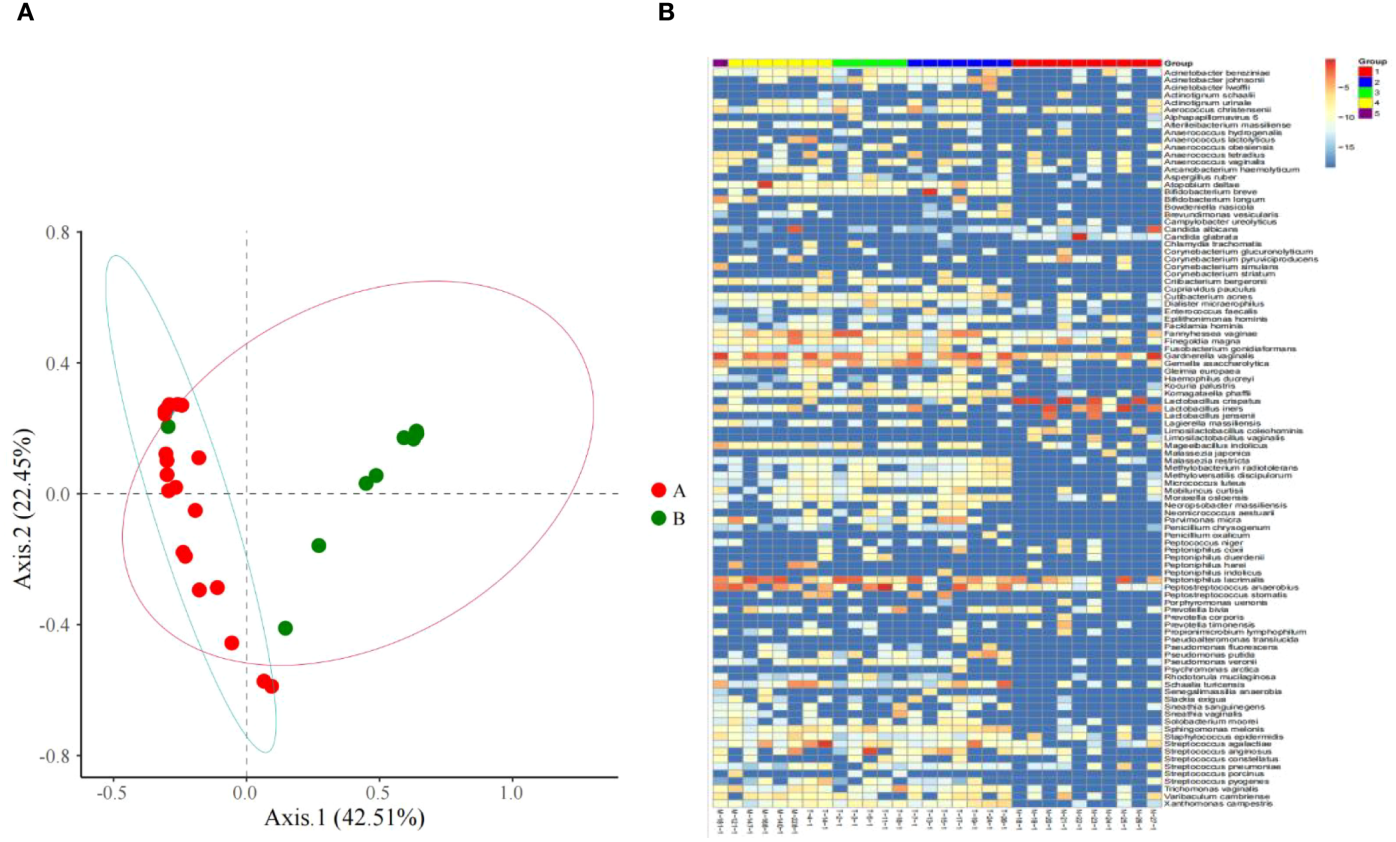
**(A)** Microbiome distribution obtained by PCoA clustering of the vaginal microbiota of all subjects; **(B)** Heat maps of vaginal microbiome abundance distribution and the distribution of microbial community.

### Shotgun metagenomic sequencing results

From the principal component analysis (PCA) plot (**Figure 1A**), marked differences in microbiota composition are observed between the two groups. Notably, one sample in the control group exhibited a microbiota profile that deviated from the intra-group cluster and aligned closely with that of the TV-infected group. This finding suggests that women clinically diagnosed as free of vaginal infection do not always present a fully normal vaginal microecological composition, subclinical infection may be present in such cases.

The species abundance differences of vaginal microbiota between women infected with Trichomonas vaginalis and those without vaginitis through different color gradients (**Figure 1B**). It can be intuitively observed that there are significant differences in the composition of vaginal microbiota between the two groups, and the relative abundances of different species show obvious distribution differences in the two group.

**Figure 2:**
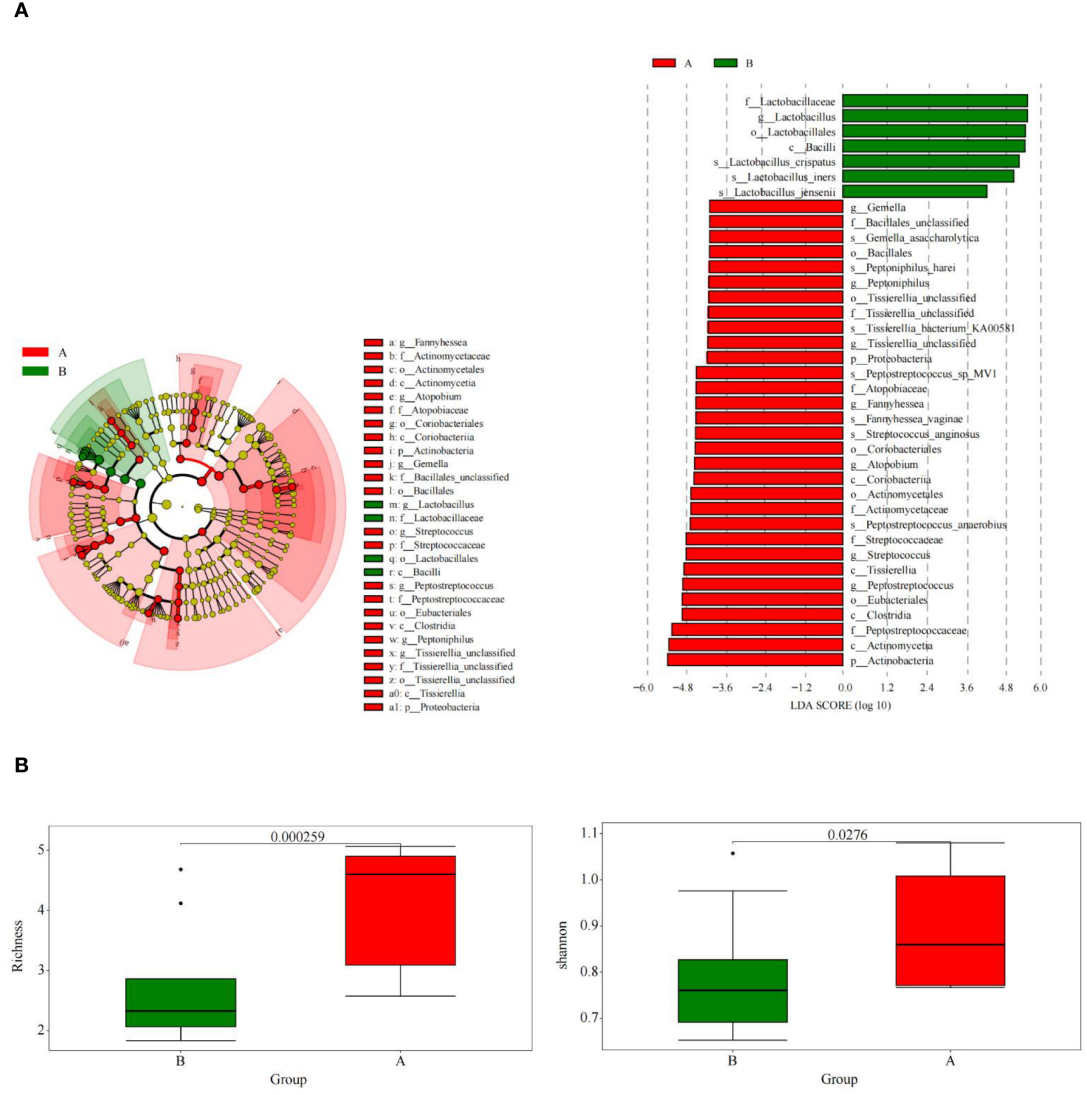
Shotgun metagenomic sequencing results. **(A)** The results of the LDA effect sizes (LEfSe), and at the species level, significant differences in microbiota were observed between the Trichomonas vaginalis (TV) infection group and the control group. **(B)** There were significant differences in species diversity and richness between the TV infection group and the control group.

This LEfSe analysis of vaginal microbiota display microbial differences at various taxonomic levels (such as genera, families, and orders), presenting the microbial taxa that differ significantly between the two groups (**Figure 2A**).

Metagenomic sequencing results showed higher α-diversity in the TV-infected group than in the uninfected control group, with higher mean species richness (**Figure 2B**, p = 0.000259, Wilcoxon) and a higher mean Shannon’s index (**Figure 2B**, p = 0.027600, Wilcoxon). Beta diversity analysis based on unweighted UniFrac distances revealed significant differences in vaginal microbial composition between the TV-infected and control groups (ANOSIM, r = 0.21, p = 0.000167, unweighted UniFrac). These community-level shifts were further supported by LEfSe analysis, which identified 38 distinguishing microbiome features with an LDA score > 3.0.

The differential taxa identified by LEfSe were validated through independent approaches: (1) Wilcoxon rank-sum tests with Benjamini-Hochberg FDR correction confirmed their statistical significance (FDR < 0.05); (2) their abundance patterns aligned with the observed broader community shifts; (3) they were consistent with microscopic findings, such as reduced Lactobacillus grades and enriched Gram-negative bacilli/anaerobic cocci.

Metagenomic sequencing revealed that the vaginal flora in TV-infected patients predominantly shifted toward anaerobic cocci and bacilli. Dominant bacteria included BV-associated anaerobic bacilli (e.g., *Gardnerella* spp., etc.), non-BV-associated anaerobic bacilli (e.g., *Bifidobacterium breve*, etc.), anaerobic cocci (e.g., *Peptostreptococcus*., etc.), and a small number of facultative anaerobic cocci (e.g., *Streptococcus agalactiae*), with a higher proportion of cocci than bacilli. Among 20 TV cases diagnosed via microscopy, metagenomic sequencing showed TV as the most abundant vaginal microorganism in only 1 case; bacteria were the most abundant in most samples. This is attributed to limitations of metagenomic sequencing in detecting eukaryotic pathogens, as standardized sampling cannot fully eliminate variability among different swabs. In 19 patients with low-load TV infection, sequencing showed low TV abundance, but all presented clinical symptoms (e.g., vaginal itching, increased discharge, and congestion), along with increased alpha diversity and altered dominant flora (e.g., reduced *Lactobacillus*, enriched anaerobic cocci/bacilli), confirming active infection even with low pathogen load (Fichorova et al., 2017; Gu et al., 2019).

**Figure 3:**
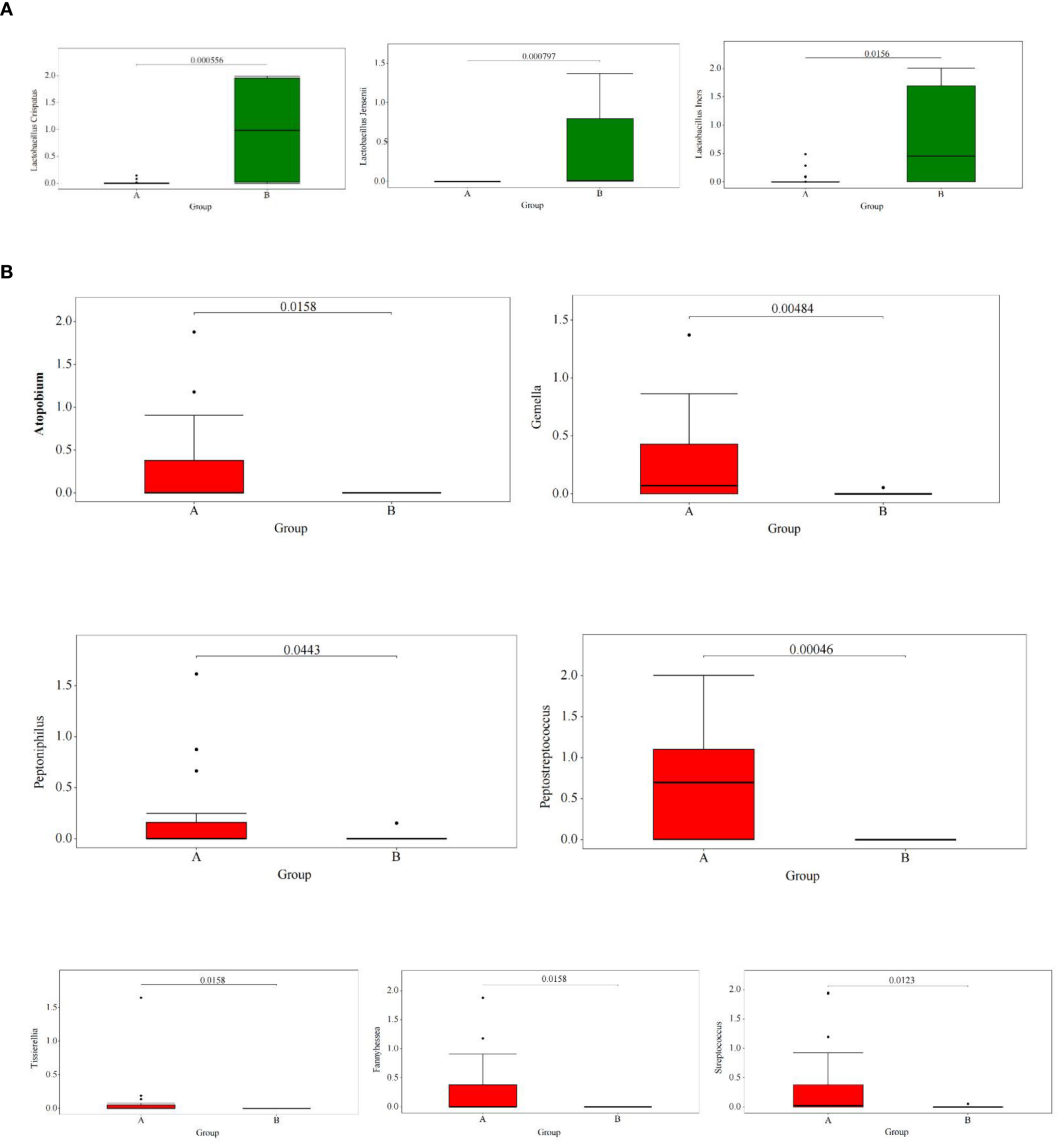
**(A)** Shotgun metagenomic sequencing results indicated the dominant microbiota in the control group at the genus level. **(B)** Shotgun metagenomic sequencing results indicated the dominant microbial species in the TV infection group at the species level.

LEfSe analysis showed that (**Figure 3**) at the genus level, compared to women in the control group, women in the TV-infected group had significantly higher abundance of *Fannyhesse* (FDR = 0.018), *Atopobium* (FDR = 0.018), *Gemella* (FDR = 0.006), *Streptococcus* (FDR = 0.014), *Peptostreptococcus* (FDR = 0.001), *Peptoniphilus* (FDR = 0.047), and *Listeria monocytogenes* (FDR = 0.018), while *Lactobacillus* was significantly lower in abundance (FDR < 0.05). At the species level, *Lactobacillus crispatus* (FDR = 0.001), *Lactobacillus iners* (FDR = 0.017), and *Lactobacillus jensenii* (FDR = 0.001) were significantly more abundant in the control group.

### Comparison of metagenomic sequencing results with microscopic examination results

**Figure 4:**
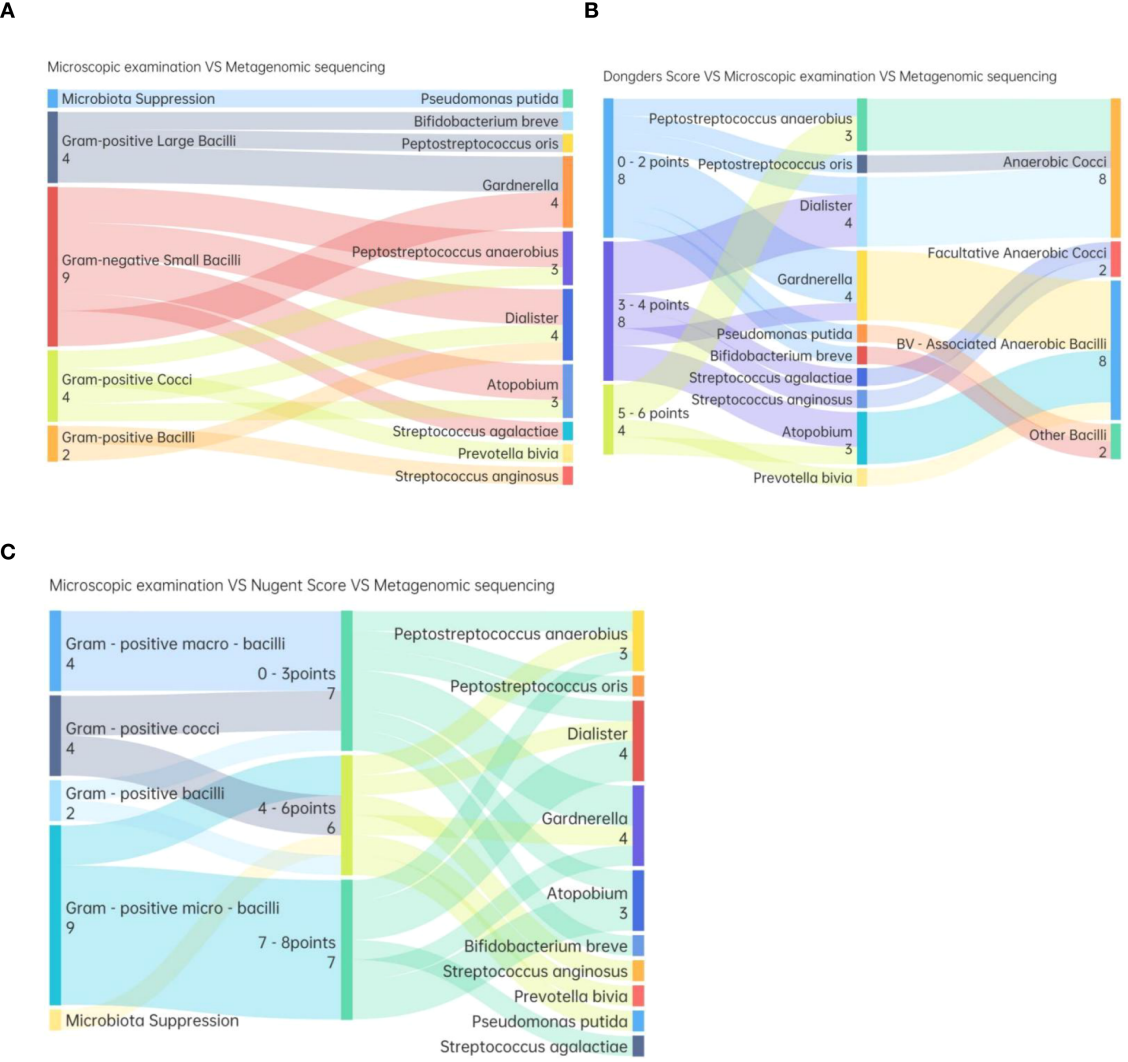
**(A)** Comparison between dominant bacteria identified by Wet-film microscopy and shotgun metagenomic sequencing results. **(B)** Comparative Analysis of Dominant Microbiota in Vaginal Discharges with Different Donders score by Wet-film Microscopy and Shotgun metagenomic Sequencing. **(C)** Comparative Analysis of Dominant Microbiota in Vaginal Discharges with Different Nugent Scores by Wet-film Microscopy and shotgun metagenomic Sequencing.

The dominant bacteria identified via microscopy in TV-infected patients differed significantly from those identified via metagenomic sequencing. Microscopy most frequently identified Gram-negative bacilli as dominant (**Figure 4**), while metagenomic sequencing revealed a high proportion of cocci (mainly *Streptococcus*, *Peptoniphilus*, *Streptococcus agalactiae*) and BV-associated bacilli (e.g., *Atopobium*, *Gardnerella*). Metagenomic sequencing most frequently identified *Gardnerella* and *Peptoniphilus* as dominant: 2 samples with dominant *Gardnerella vaginalis* were identified as Gram-negative small bacilli via microscopy, while another 2 were identified as Gram-positive large bacilli. Among 4 samples with dominant *Peptoniphilus*, microscopy identified 2 as Gram-negative small bacilli, 1 as Gram-positive cocci, and 1 as Gram-positive bacilli. In TV-infected samples where microscopy identified Gram-positive large bacilli as dominant, metagenomic sequencing revealed dominant *Bifidobacterium breve*, *Peptostreptococcus stomatis*, and *Gardnerella vaginalis*.

**Table 1:** Comparison of vaginal symptoms and microecological conditions between two groups of women.

Characteristics	Total number	V group	Control group	T value	P - value
	(n=30)	(n=20)	(n=10)		
Vaginal microbiota Density Absent - I/II - III/IV	1/28/1	1/18/1	0/10/0		1.000/0.540/1.000
Vaginal microbiota Diversity Absent - I/II - III	15/15	5/15	7/3		0.045
Dominant Bacteria Absent/Gram-positive Large Bacilli/Gram- positive Bacilli/Gram-positive Cocci/ Gram-negative Small Bacilli	1/14/3/3/9	1/4/3/3/9	0/10/0/0/0		1.000/0.000/0.532/0.532/0.013^*^
Lactobacillus Grade (I - IIa/IIb - III)	15/15	5/15	10/0		0.000^*^
pH		4.715 ± 0.67	4.07 ± 0.095	3.003	0.000^*^
Nugent Score 0-3/4-6/7-8	17/6/7	7/6/7	10/0/0		0.001^*^/0.074/0.064
Donders Score 0-2/3-4/5-6	18/8/4	8/8/4	10/0/0		0.002^*^/0.029^*^/0.272
Age		39.450 ± 13.873	38.100 ± 10.225	0.272	0.788

*Fisher’s exact test

Metagenomic sequencing of controls (with Gram-positive large bacilli as dominant vaginal microbiota) showed most dominant bacteria were *Lactobacillus* (5 cases of *Lactobacillus crispatus*, 3 cases of *Lactobacillus iners*, 1 case of *Malassezia japonica*, 1 case of *Gardnerella vaginalis*).

### TV co-infection with AV

All 20 TV-infected patients had vaginal wall congestion or increased yellow discharge, and 12 patients had a Donders score ≥ 3. **Table 2** shows Donders scores in the experimental group compared with the results of metagenomic sequencing. High-throughput showed that patients with Donders scores ≥3 had dominant anaerobic cocci and bacilli, with only 2 cases (10%) dominated by facultative anaerobic cocci.

**Table 2:** Patients with Trichomoniasis vaginalis are co-infected with Candida vulvovaginal.

No	Type	Microscopic examination for Fungi	Nugent score	Bacterial community diversity	Donders score	Dominant bacteria of metagenomic analysis	Dominant bacteria of microscopic
1	Candida albicans	Detected	5	3	2	Gardnerella vaginalis	Gram negative bacilli(s)
2	Candida albicans	Not detected	8	3	3	Fannyhessea vaginae	Gram negative bacilli(s)

Patients diagnosed with TV+AV infection, their dominant microbiota were *Peptostreptococcus anaerobius, Fannyhessea vaginae, Streptococcus anginosus, Peptoniphilus lacrimalis, and Gemella asaccharolytica.*

**Table 3:** TV and BV coinfection: diagnostic agreement evaluation.

Microscopic examination (nugent score)	Metagenomic sequencing		Total
	Co-infected with BV	Not Co-infected with BV	
Co-infected with BV	5	8	13
Not Co-infected with BV	3	4	7
Total	8	12	20

Guideline-recommended molecular diagnosis criterion of AV with reduced Lactobacillus load and a ≥ 10-fold increase in aerobic bacteria (Cooperative Group of Infectious Disease, 2021b). However, metagenomic sequencing results did not support a TV+AV diagnosis in 80% of the 5 cases diagnosed with TV+AV via clinical criteria (**Figure 4B**).

### TV co-infection with BV

Nugent score of Gram-stained smears in TV-infected patients identified BV co-infection in 35% (7/20) of cases, all of which with Donders scores ≥co Metagenomic sequencing showed dominant cocci (4/7) and bacilli (3/7) in TV+BV patients, versus dominant cocci (3/7) and bacilli (4/7) in non-BV patients (Nugent score 0–3), with no significant difference in distribution. TV-infected patients had vaginal microbiota dominated by anaerobic cocci and bacilli, with most anaerobic bacilli being BV-related. Although Peptostreptococcus often proliferates in BV, anaerobic bacilli overgrowth is the main floral pattern in BV (**Figure 4C**).

Using metagenomic sequencing detection of *Gardnerella*, *Prevotella*, and *Atopobium* as dominant bacteria for BV diagnosis, and Nugent score ≥7 (microscopy) as the BV criterion, the kappa value was -0.038, indicating poor agreement between the two methods for diagnosing TV+BV co-infection (**Table 3**).

### TV co-infection with VVC

Microscopy diagnosed 1 case of VVC, while metagenomic sequencing identified 2 cases: 1 case of Candida albicans (43.47% abundance, not detected by microscopy) and 1 case of Candida albicans (0.19% abundance, detected by microscopy) **Table 2**.

### CST typing in TV-infected patients

Ravel et al. categorized female vaginal microbiome into CST I–V: CST I (Lactobacillus crispatus-dominant), CST II (*Lactobacillus gasseri*-dominant), CST III (*Lactobacillus iners*-dominant), CST V (*Lactobacillus jensenii*-dominant), and CST IV (mixed microbiota) (Ravel et al., 2010), further subdivided into CST-IV-A (high abundance of *Ca. L. vaginalis*, moderate abundance of *G. vaginalis* and *Atopobium vaginae*), CST-IV-B (high abundance of *G. vaginalis*, low abundance of *Ca. L. vaginae*, and moderate abundance of *Atopobium vaginae*), and CST-IV-C (subdivided into 5 categories: CST IV-C0 with moderate *Prevotella*; CST IV-C1 with dominant *Streptococcus*; CST IV-C2 with dominant *Enterococcus*; CST IV-C3 with dominant *Bifidobacterium*; CST IV-C4 with dominant *Staphylococcus*) (France et al., 2020). All TV-infected patients in this study had CST IV microbiota: 7 cases of CST IV-B, 12 cases of CST IV-C, and 1 case of microbiota suppression. Among CST IV-C cases, there were 6 cases of IV-C1, 4 cases of IV-C2, and 1 case each of IV-C0 and IV-C3. No Ca. L. vaginalis was detected in TV-infected patients (**Table 4**).

**Table 4:** Changes in vaginal dominant flora among TV infection patients using different detection methods.

No.	Diagnose by microscopy	Grouping of microscopic examination results	Bacterial community diversity	Bacterial community density bacterial community density	Dominant bacteria of microscopic	Dominant bacteria of metagenomic analysis	Relative abundance	CST classification	CST subtype
N-18-1	Normal	Normal Flora	1	3	Gram-positive Large bacilli	Lactobacillus crispatus	88.5	I	I-A
N-19-1	Normal	Normal Flora	1	3	Gram-positive Large bacilli	Lactobacillus crispatus	97.1	I	I-A
N-20-1	Normal	Normal Flora	2	3	Gram-positive Large bacilli	Lactobacillus iners	70.5	III	III-B
N-21-1	Normal	Normal Flora	2	3	Gram-positive Large bacilli	Lactobacillus crispatus	84.9	1	I-A
N-22-1	Normal	Normal Flora	1	3	Gram-positive Large bacilli	Lactobacillus iners	93.2	III	III-A
N-23-1	Normal	Normal Flora	1	3	Gram-positive Large bacilli	Lactobacillus crispatus	43.6	I	I-B
N-24-1	Normal	Normal Flora	2	3	Gram-positive Large bacilli	Malassezia japonica	52	IV	
N-25-1	Normal	Normal Flora	1	3	Gram-positive Large bacilli	Lactobacillus iners	64.1	III	III-B
N-26-1	Normal	Normal Flora	1	3	Gram-positive Large bacilli	Lactobacillus crispatus	98.7	I	I-A
N-27-1	Normal	Normal Flora	1	3	Gram-positive Large bacilli	Gardnerella vaginalis	83.66	IV	IV-B
M-121-01	TV+AV+BV	Abnormal Flora BV type	2	2	Gram-negative Small bacilli	Peptostreptococcus anaerobius	14.1	IV	IV-C1
M-140-01	TV+AV+BV	Abnormal Flora BV type	3	3	Gram-negative Small bacilli	Peptoniphilus lacrimalis	23.8	IV	IV-C2
M-147-01	TV+AV+BV	Abnormal Flora BV type	4	3	Gram-negative Small bacilli	Atopobium deltae	27.3	IV	IV-C2
M-168-01	TV+AV+BV	Abnormal Flora BV type	3	2	Gram-negative Small bacilli	Peptoniphilus lacrimalis	46.3	IV	IV-B
M-181-01	TV+VVC	Abnormal Flora BV type	3	3	Gram-negative Small bacilli	Gardnerella vaginalis	36.1	IV	IV-B
M-228-01	TV+AV+BV	Abnormal Flora BV type	3	3	Gram-negative Small bacilli	Fannyhessea vaginae	8.3	IV	IV-B
T-11-01	TV+AV,	Abnormal Flora non-bv type	3	2	Gram-positive cocci	Peptostreptococcus anaerobius	64.3	IV	IV-C1
T-13-01	TV	Normal Flora	3	2	Gram-positive Large bacilli	Bifidobacterium breve	51.3	IV	IV-C3
T-14-01	TV+AV+BV	Abnormal Flora BV type	3	3	Gram-negative Small bacilli	Streptococcus agalactiae	49.1	IV	IV-C1
T-15-01	TV	Normal Flora	3	2	Gram-positive Large bacilli	Peptostreptococcus stomatis	2.6	IV	IV-C1
T-17-01	TV	Abnormal Flora BV type	3	3	Gram-negative Small bacilli	Peptostreptococcus anaerobius	15.9	IV	IV-C1
T-18-01	TV+AV	Abnormal Flora non-bv type	2	1	Gram-positive cocci	Trichomonas vaginalis	1.8	IV	IV-C0
T-19-01	TV	Normal Flora	3	1	Gram-positive Large bacilli	Gardnerella vaginalis	23.5	IV	IV-B
T-2-1	TV+AV	Abnormal Flora non-bv type	2	1	Gram-positive cocci	Peptoniphilus lacrimalis	19.1	IV	IV-C2
T-24-01	TV	Flora deficiency	0	0	Non dominant bacteria	Pseudomonas putida	1.9	IV	IV-B
T-26-01	TV	Normal Flora	3	1	Gram-positive Large bacilli	Gardnerella vaginalis	16.5	IV	IV-B
T-3-01	TV+AV	Abnormal Flora non-bv type	2	2	Gram-positive cocci	Fannyhessea vaginae	16.6	IV	IV-B
T-4-1	TV+AV+BV	Abnormal Flora BV type	2	2	Gram-negative Small bacilli	Gardnerella vaginalis	14.1	IV	IV-B
T-5-1	TV+AV	Abnormal Flora non-bv type	3	2	Gram-positive bacilli	Streptococcus anginosus	29.2	IV	IV-C1
T-7-1	TV	Abnormal Flora non-bv type	2	2	Gram-positive bacilli	Peptoniphilus lacrimalis	14.6	IV	IV-C2

## Discussion

### Vaginal microbiome analysis in TV-infected patients

This study used different swabs for metagenomic sequencing and microscopic examination, but all samples were collected from the same anatomical site (upper 1/3 of the posterior vaginal fornix) following standardized protocols (e.g., no vaginal manipulation 3 days prior, uniform swab type and sampling method), minimizing sampling bias from spatial heterogeneity and operational variability. The two methods showed partial correlation in reflecting vaginal microecology: dominant genera identified by metagenomic sequencing (e.g., *Gardnerella, Peptoniphilus*) partially corresponded to microscopic observations of Gram-negative small bacilli and Gram-positive cocci; the predominantly CST IV classification aligned with the overall trend of reduced *Lactobacillus* and increased anaerobic bacteria observed microscopically, indicating good sample homology. Discrepancies (e.g., differential detection of low-abundance Candida) were attributed to methodological differences (morphological identification vs. gene sequencing) rather than sampling bias, suggesting minimal impact of using different swabs.

Analysis of vaginal microbiome composition and diversity in TV-infected patients showed significantly higher microbiota diversity, Nugent scores, and AV scores compared to controls (p < 0.05), indicating more complex vaginal microecological changes in TV infection. Metagenomic sequencing analysis of α/β diversity and LEfSe confirmed significantly higher species richness and distinct microbial composition in TV-infected women versus controls. TV-infected patients had significantly higher vaginal pH (mean 4.715 ± 0.67), consistent with (McGrory et al., 1994), likely due to reduced *Lactobacillus* (which maintains low pH via acidic metabolites), creating a favorable environment for TV growth.

Among clinically diagnosed TV cases, metagenomic sequencing showed high TV abundance in only 1 case; the remaining had low abundance but presented clinical symptoms, indicating that low-abundance TV infections can cause clinical manifestations.

Metagenomic sequencing revealed that TV-infected patients lacked *Lactobacillus* and had increased anaerobic cocci, with significant differences from controls at both genus and species levels: increased *Fannyhessea, Atopobium, Gemella, Streptococcus, Peptoniphilus, Listeria monocytogenes, and Peptostreptococcus*; controls were dominated by *Lactobacillus crispatus, Lactobacillus iners, and Lactobacillus jensenii*. High abundances of *Fannyhessea vaginae* and *Streptococcus* are associated with increased cervical lesion risk, and *Fannyhessea vaginae* is strongly linked to BV (Gerson et al., 2020). In contrast, *Lactobacillus crispatus* maintains mucosal integrity and reduces genital infection risk; *Lactobacillus iners* survives across a wide pH range and under metabolic stress; *Lactobacillus jensenii*, abundant in healthy women, has antimicrobial and immunomodulatory properties. These findings suggest that TV-infected women almost universally have vaginal microbiota dysbiosis, which may induce other vaginal infections and increase cervical lesion risk.

Metagenomic sequencing identified *Gardnerella* and *Peptoniphilus* as the most frequent dominant bacteria, indicating that anaerobic bacilli and cocci overgrowth are the main patterns in TV infection. The Nugent score, commonly used to assess anaerobic bacilli (e.g., *Mobiluncus*) overgrowth via microscopy, is less effective in TV-infected patients due to increased bacterial diversity. Microscopy-dominant bacteria poorly describe mixed vaginitis microbiota, especially when diversity is grade 3 or higher, indicating complex microbiota overgrowth.

### Vaginal microbiome analysis, diagnosis, and treatment of TV-related mixed vaginitis

According to the ACOG Practice Bulletin on Vaginitis Management in Nonpregnant Patients (Vaginitis in Nonpregnant Patients: ACOG Practice Bulletin, Number 215, 2020) and CDC Trichomoniasis Treatment Guidelines (Workowski et al., 2021), nucleic acid amplification tests (NAAT) should complement microscopy for pathogens like TV, particularly in low-load, asymptomatic, or clinically suspected cases with negative traditional tests, to improve diagnostic sensitivity. In addition, screening for HIV-infected women should be emphasized, the association between pregnancy vaginitis and adverse outcomes evaluated, and standardized treatment (including partner treatment for TV) implemented.

Research on mixed vaginitis clinical manifestations, pathogenesis, diagnosis, and treatment is limited (Benyas and Sobel, 2022). Mixed vaginitis involves two or more pathogens causing symptoms and signs. Epidemiological data suggest TV often coexists with BV, but both may be underestimated due to asymptomatic presentations and overlapping features (e.g., discharge, inflammation) (Fichorova et al., 2017). BV is characterized by reduced *Lactobacillus* and anaerobic overgrowth. *Gardnerella vaginalis* (present in 95% of BV cases) adheres to vaginal epithelium, forming a biofilm that supports other microorganisms (e.g., *Atopobium, Prevotella, Bacteroides, Mycoplasma*) (Onderdonk et al., 2016). This study found significantly higher Nugent scores in TV-infected patients versus controls, but poor concordance between Nugent scores and high-throughput sequencing. Many patients diagnosed with BV via Nugent score showed extensive anaerobic cocci overgrowth with minimal anaerobic bacilli proliferation, highlighting limitations of the Nugent score (which focuses on anaerobic bacilli) in diagnosing TV+BV co-infection.

The poor diagnostic consistency between microscopic examination and high-throughput sequencing for vaginal mixed infections carries significant clinical implications. Firstly, Traditional microscopy is limited in identifying complex mixed infections due to reliance on morphological recognition, often missing low-abundance, atypical, or non-culturable pathogens, leading to underdiagnosis/misdiagnosis and inappropriate treatment. Secondly, high-throughput sequencing, which comprehensively profiles microbial communities (including anaerobic cocci, rare fungi, and opportunistic pathogens), better reflects microecological imbalance in mixed infections, supporting its use in refractory/recurrent cases. Thirdly, inadequate diagnosis may lead to persistent inflammation, recurrence, or complications (e.g., increased cervical lesion or STI risk) due to synergistic pathogen interactions (e.g., TV promoting anaerobic overgrowth).

AV infection is common, research showed higher mixed infection rates than single AV (Fan et al., 2012). Currently, AV is diagnosed by Donders score in combination with the clinical symptoms (vaginal wall congestion, yellow discharge)—symptoms also seen in TV infection. All 20 TV-infected patients had these symptoms, with 12 having Donders scores ≥3, raising concerns about the accuracy of TV+AV diagnosis via clinical criteria.

Zhu et al. reported AV+BV, AV+VVC, and AV+TV as the most common mixed AV infections (Zhu et al., 2014), characterized by *Lactobacillus* absence, severe inflammation (worse than BV), and aerobic cocci presence (Zhang and Qin, 2017). In this study, a total of 12 AV infection cases (60%) diagnosed by Donders score, but metagenomic sequencing showed 2 cases with dominant facultative anaerobes (*Streptococcus agalactiae, Streptococcus anginosus*) and 10 cases with dominant anaerobes, all with significantly reduced *Lactobacillus*. Over 80% of these cases would not be diagnosed with AV via metagenomic sequencing, indicating high bias in using Donders scores to diagnose TV-associated mixed AV infections.

TV+VVC co-infection is rare, and in this study metagenomic sequencing identified 2 Candida albicans cases among 20 TV-infected patients, with microscopy detecting only 1. One sample met BV criteria via Nugent score and sequencing, with an AV score of 3 and symptoms, suggesting possible TV+BV+VVC+AV co-infection. In multiple mixed infections, interactions between factors complicate vaginal microecology, affecting microscopic judgment. Metagenomic sequencing is more sensitive for fungal detection, identifying low-load fungi missed by microscopy, which relies on morphological observation and may miss small quantities, atypical forms, or complex microecological changes.

Mixed vaginitis remains underexplored in the literature. Pathogens in mixed infections may synergize, alter microbiome, and exhibit distinct characteristics. This study found that clinically diagnosed AV (via Donders score and symptoms) was not supported by metagenomic sequencing, suggesting that TV-induced anaerobic environments inhibit AV. Moreover, only Donders scores/symptoms are inaccurate for mixed AV diagnosis—highlighting metagenomic sequencing as a precise auxiliary tool. For TV+BV co-infection, poor concordance between Nugent scores and high-throughput sequencing indicates limitations of Nugent scoring in this context.

In this study, it was observed that microscopy poorly describes microbiota changes in mixed infections, while metagenomic sequencing more accurately characterizes mixed vaginitis microbiota with greater depth and breadth, particularly when contamination was minimized and an appropriate extraction method was employed (Quince et al., 2017). Inconsistencies between methods suggest TV infection induces complex microbiota changes unrecognizable by traditional microscopy, limiting its ability to assess complex microbial overgrowth. Metagenomic sequencing identifies species via gene sequences, detects low-abundance pathogens, and enables comprehensive understanding of microbiota structure. Current clinical diagnosis relies on morphological and functional microecological evaluations; thus, rapid, accurate analysis of complex mixed vaginitis microbiota remains a critical research gap.

Vaginitis treatment aims to restore normal microecology rather than merely eliminating pathogens. Traditional TV treatment uses nitroimidazoles, with variable efficacy against anaerobes. For TV-related mixed infections, treatment should target TV first, with additional drugs for other pathogens (e.g., anti-anaerobes for BV, antifungals for VVC). This study found increased *Gardnerella* in TV-infected patients; previous research showed 63.33% of BV-related *Gardnerella* are metronidazole-resistant (Fan et al., 2021). While nitroimidazoles often eliminate most vaginal trichomonas, they may not restore normal flora. Post-treatment anaerobic overgrowth requires clinical attention and further research (Molina et al., 2022; Waetjen et al., 2023).

### CST types in TV-infected patients

Ravel et al. classified reproductive-aged women’s vaginal microbiota into 5 CSTs, further subdivided into 11 subtypes (France et al., 2020). One study evaluated the association between different CST state types and TV and found that 72% of TV-infected women had CST-IV microbiota (Brotman et al., 2012). In this study, all TV-infected patients had CST IV microbiota: 7 cases of IV-A/B, 12 cases of IV-C, and 1 case of microbiota suppression, including 6 IV-C1, 4 IV-C2, 1 IV-C0, and 1 IV-C3. CST IV indicates reduced *Lactobacillus* (*L. crispatus, L. iners, L. jensenii*) and overgrowth of other bacteria in TV-infected women, damaging mucosal integrity, altering pH, and disrupting healthy microbiota.

TV-infected patients predominantly had CST IV-C. CST IV may increase TV infection risk, and TV may alter microbiota—CST IV bacteria and TV synergistically disrupt vaginal epithelial tight junctions and paracellular permeability, compromising the cervicovaginal barrier and increasing risks of other vaginal inflammations and cervical lesions. CST IV-C is common in postmenopausal women; IV-C1 is associated with vaginal pain. CST IV may increase BV and cervical lesion risks (Fong Amaris et al., 2024). For pregnant women, CST IV is linked to mid-pregnancy cervical shortening (Gerson et al., 2020), and preterm labor in African Americans (possibly related to high CST IV prevalence) (Fettweis et al., 2019). CST IV both increases TV risk and synergizes with TV, reflecting microbiota health and changes Compared to microscopy, CST typing comprehensively analyzes microbiota at the community level, considers *Lactobacillus* and other bacteria abundances, predicts disease risk, dynamically monitors microbiota changes during disease, and reflects microenvironmental shifts—providing clearer categorization of TV-related microbiota.

However, differences in clustering algorithms, sequencing regions, and threshold definitions for dominant bacteria among various studies hinder cross-study CST comparisons. CST relies solely on microbial composition, excluding host factors (genetics, immunity, hormones), and cannot directly guide treatment, requiring integration with symptoms, signs, and tests (e.g., pH, amine test) to avoid overreliance. CST typing may misclassify ambiguous samples due to forced categorization. To address this, probabilistic clustering algorithms can be adopted to calculate the probability of a sample belonging to each CST. Meanwhile, a comprehensive judgment should be made in combination with clinical phenotypes, and longitudinal dynamic monitoring can be conducted when conditions permit.

## Conclusion

This study identifies differences in microbial composition between TV-infected women and healthy women with Gram-positive large bacilli as dominant flora. TV infection induces vaginal microecological imbalance, increasing risks of other vaginal infections and cervicitis. TV-infected patients typically have reduced Lactobacillus, anaerobic overgrowth, and mixed infections, leading to complex flora changes. Nugent scores, AV scores, and microscopic detection of hyphae/spores have limitations in diagnosing mixed vaginal infections. Microscopy has high missed diagnosis and misdiagnosis rates in mixed vaginitis, while metagenomic sequencing identifies more key pathogens and comprehensively assesses microbiota structure. CST typing better describes vaginal microecology, with TV patients predominantly having CST-IV-C. However, sequencing is costly and time-consuming; integrating it into clinical practice requires further research.

we propose that the diagnosis of vaginitis should follow a stepwise diagnostic way, where microscopic examinations (wet mount, Gram staining) serve as the first-line method for initial screening. However, due to their inherent limitations, molecular diagnostic approaches (NAAT, high-throughput sequencing) should be used as complementary tools for precise analysis in cases of recurrent vaginitis, treatment failure (e.g., persistent symptoms following nitroimidazole therapy for Trichomonas vaginalis), or suspected polymicrobial infections. These advanced techniques can guide therapeutic adjustments and enable prediction of associated pathologies through CST (community-state types) classification.

To address the challenges of time-consuming and high cost associated with metagenomic sequencing, promising directions for further research include the development of targeted metagenomics (sequencing only pathogen-specific genes rather than entire genomes) and pathogen-specific detection kits (e.g., multiplex PCR assays for *Fannyhessea* and *Gardnerella*).

## Data Availability

The datasets presented in this study can be found in online repositories. The names of the repository/repositories and accession number(s) can be found in the article/supplementary material.
